# Relationship between arterial stiffness, left ventricular diastolic function, and renal function in chronic kidney disease

**DOI:** 10.1186/s12882-023-03308-w

**Published:** 2023-09-03

**Authors:** Balázs Sági, István Késői, Tibor Vas, Botond Csiky, Judit Nagy, Tibor József Kovács

**Affiliations:** 1 2nd Dept. of Internal Medicine and Nephrology, Diabetes Centre, Medical School, Clinical Center, University of Pécs, Pacsirta Street 1, Pécs, 7624 Hungary; 2 Mohács Hospital, Department of Internal Medicine Cardiology, Szepessy square 7, Mohács, 7700 Hungary

**Keywords:** Arterial stiffness, Chronic kidney disease, Echocardiography, Doppler, IgA nephropathy, Left ventricular diastolic dysfunction

## Abstract

**Aim:**

In chronic kidney disease, IgA nephropathy, and left ventricular diastolic dysfunction have prognostic significance as well. However, the relationship between diastolic dysfunction, arterial stiffness, and renal function has not been fully elucidated.

**Methods:**

79 IgA nephropathy patients (aged 46 ± 11 years) and 50 controls were investigated. Tissue Doppler imaging was used to measure early (Ea) and late (Aa) diastolic velocities. Arterial stiffness was measured by a photoplethysmographic (stiffness index (SI)) and an oscillometric method (aortic pulse wave velocity (PWVao)).

**Results:**

We compared the IgAN patients to a similar cardiovascular risk group with a preserved eGFR. A strong correlation was found between Ea/Aa and SI (p < 0.001), also with PWVao (p < 0.001), just in IgAN, and with eGFR (p < 0.001) in both groups. IgAN patients were divided into groups CKD1-2 vs. CKD3-5. In the CKD 3–5 group, the incidence of diastolic dysfunction increased significantly: 39% vs. 72% (p = 0.003). Left ventricle rigidity (LVR) was calculated, which showed a close correlation with SI (p = 0.009) and eGFR (p = 0.038). By linear regression analysis, the independent predictors of SI were age, E/A, and E/Ea; SI was the predictor of LVR; and E/A and hypertension were the predictors of eGFR.

**Conclusion:**

In chronic kidney disease, increased cardiac rigidity and vascular stiffness coexist with decreased renal function, which is directly connected to diastolic dysfunction and vascular stiffness. On the basis of comparing the CKD group to the control group, vascular alterations in very early CKD can be identified.

## Summary at a glance

In our study, we examined patients with IgA nephropathy, a chronic kidney disease, to identify those at high cardiovascular risk.

Arterial stiffness and tissue Doppler echocardiographic parameters showed a significant association with kidney function; therefore, a common pathological vascular and myocardial remodeling can be assumed.

## Introduction

Former large-scale studies revealed that CV mortality and morbidity in chronic kidney disease (CKD) are many times higher than in the general population, posing a global public health problem [1, 2]. IgA nephropathy (IgAN) is the most common immunocomplex-mediated primary glomerular disease all over the world. Patients with IgAN tend to progress, and therefore about half of the patient’s kidney disease deteriorates into ESRD within 15 years [3]. Arterial stiffness has been proposed as a risk factor for future cardiovascular events [4–7]. Pulse wave velocity (PWV) is simple, validated, and widely used to measure arterial stiffness in research and clinical practice [8]. Recent studies have shown that arterial stiffness has an important role in left ventricular (LV) diastolic function [9–12]. LV diastolic dysfunction appears to be associated with LV hypertrophy and reduced coronary perfusion, facilitated by increased arterial stiffness [12, 13]. The remodeling of the myocardium and blood vessels could be one of these risk factors leading to CV events, heart failure, and further progression to end-stage renal disease (ESRD) in CKD as IgAN. In fact, eGFR (estimated glomerular filtration rate) is an important determinant of both target organ damage [14]. Therefore, identifying these risk factors and high-risk patients is very important for interventional strategies and managing patients with CKD.

Echocardiography is a widely used, valuable, noninvasive method for the determination of the left ventricular systolic and diastolic function (LVDD), which has prognostic significance in ischemic heart disease, heart failure, and end-stage renal failure [15–18]. Tissue Doppler Imaging (TDI) echocardiography is another way to measure the rate of myocardial contractility and helps refine diastolic dysfunction. In community-based epidemiological studies, the ratio of E (transmitral E wave velocity) to Ea (early diastolic mitral velocity) has been reported to be significantly associated with LV diastolic function and filling pressure [19]. A study published a decade ago found that renal dysfunction is associated with worse outcomes and higher mortality in HFpEF patients [20]. Despite the association between CKD and adverse outcomes, the interaction between CKD, clinical features, and cardiac structural and functional abnormalities in HFpEF has not been fully understood. There could be differences in arterial stiffness between the different etiological groups of CKD with similar renal function, as we demonstrated in our previous study [21].

This study was performed to investigate the association between LV diastolic function and arterial stiffness in relation to renal function in a homogenous group of CKD patients with IgAN and compare it with a similar healthy group. Our hypothesis was that the association between arterial stiffness and LV diastolic function could be more pronounced in patients with deteriorated renal function.

## Methods

### Patients

Seventy-nine patients with IgAN and fifty control patients with a relatively similar, moderate CV risk were involved in this study from the 2nd Department of Internal Medicine, Nephrology, and Diabetes Center of the Clinical Center at the University of Pécs. The diagnosis of IgAN was confirmed by renal biopsy in all patients. The patients in the control group had preserved renal function and were also outpatients of our clinic with relatively similar cardiovascular risk. The local ethics committee approved the clinical study protocol (no. 3170/2008), and written informed consent was obtained from all participants.

In this cross-sectional study, echocardiography was performed, and classic CV risk factors (hypertension, diabetes, obesity, lipid abnormalities, smoking) and patient medication were also recorded. The metabolic syndrome was defined according to the ATP III (Adult Treatment Panel III) criteria. The obesity criteria were a BMI over 30 kg/m2. The CKD-EPI formula was used to estimate renal function (eGFR, ml/min, 1.73 m²). Patients with acute or chronic severe comorbidities (malignancies requiring active treatment, fever, and kidney transplant patients) were excluded. Renal replacement therapy or a history of kidney transplantation were also exclusion criteria. A Meditech ABPM (Meditech Ltd., Hungary) device was used to determine the patient’s 24-hour average systolic and diastolic blood pressure, pulse pressure, and diurnal index. Additional CV examinations (ergometry, coronagraphy, etc.) were also performed based on the patient’s complaints. The atherosclerotic cardiovascular disease (ASCVD) risk score was calculated by the American College of Cardiology ASCVD Risk Estimator Plus calculator.

### Measurement of arterial stiffness

In this study, we used the finger photoplethysmography method by the Pulse Trace System (Micro Medical Ltd., Rochester, UK) to assess pulse-wave velocity (PWV) [20, 22]. This method enables one to determine the stiffness index (SI), which can be derived from the DVP and is reflected as SI_DVP_. The DVP includes two distinct waves during the cardiac cycle: the early systolic one that originates from the pressure wave at the time of the left ventricle ejection, which could be measured in the finger artery, followed by the second peak due to a reflected wave from the more peripheral segments, usually the aortic bifurcation. The SI is derived from the body height relative to the time difference between the forward and reflective pulse waves: SI_DVP_ (m/s) = height/t. The recorded pulse curve profile is principally determined by the PWV of the large arteries [23–25]. Based on the literature data, the method used here does correlate with other methods, such as the central aortic PWV [24]. Higher SI_DVP_ values indicate increased vascular stiffness [26–28]. The central (aortic) systolic blood pressure (aoSBP) and aortic pulse wave velocity (aoPWV) were measured by the Tensiomed Arteriograph (Meditech Ltd., Hungary), which is a validated oscillometric technique to measure arterial stiffness [29].

Measurements were performed between 9:00 and 11:00 in the morning. Subjects were studied in the supine position after approximately 10 min of rest in a temperature-controlled and quiet environment. Patients were allowed to take their regular medications, but smoking, alcohol drinking, and caffeine consumption were prohibited on the day of examination. A single waveform was obtained by averaging the DVP profile for 30 s. To enhance the accuracy of the SI_DVP_ measurements, five-period samples were taken, and the upper and lower merits of DVP were deleted. The remaining three merits were averaged and used for further analyses, including the variability test. All measurements were made by the same experienced operator, blinded to all clinical data. The intraobserver coefficient of variation for SI was approximately 5.0% in our laboratory.

### Echocardiographic measurement

An Aloka SSD 1400 echocardiography machine was used. Using 2D images of the length of the apical left ventricular segment and the area of the left ventricular short-axis muscle, the left ventricular mass (LVM) was calculated (LVM = (5/6 area * length)). The Cornell criterion was used to determine LVMI, and the result was indexed for height (in meters). The unidirectional Simpson method was used to determine the diastolic and systolic left ventricular volumes, providing the left ventricular ejection fraction (LVEF): EF = ((Dvol-Svol)/Dvol)*100. Based on standard spectral Doppler measurements, mitral inflow and pulmonary venous flow were used to evaluate diastolic function. We also calculated the isovolumetric relaxation time (IVRT), the E wave deceleration time, and the E wave to A wave ratio (E/A ratio). LVH was defined as abnormal RWT and/or LVMI. TDI was used to measure the early and late displacements of the lateral basal wall fragment closest to the left ventricle (Ea and Aa) and determine the E/Ea and Ea/Aa ratios. To exclude interindividual differences, two investigators examined all patients.

### Statistical analysis

For statistical analysis, we divided our patients into two groups according to eGFR (CKD 1–2 vs. CKD 3–5). All values, unless otherwise stated, are mean ± SD. Differences between the two groups were compared by the Student’s t-test and Mann-Whitney U test for continuous variables and χ^2^ test for categorical variables. The relationship between two continuous variables was assessed by a bivariate correlation method (Pearson’s correlation), and nonparametric variables were assessed by Spearman’s correlation. The factors that influence Ea and Ea/Aa were investigated using univariate and multivariate linear regression analysis. We also used linear regression analysis to assess the associations between Ea and other covariates. SPSS version 22.0 (Statistical Program for Social Sciences for Windows, SPSS Inc., Chicago, IL) was used to analyze the data, and a statistically significant value of 0.05 was used.

## Results

Baseline clinical data are shown in Table 1. We compared the IgAN patients to a preserved renal function control group, and we found a significant difference in average blood pressure, the incidence of hypertension, eGFR, SI, PWVao, Aix, MAU, and the use of ACE inhibitors. There was no significant difference between the two groups in age, gender, other metabolic parameters (diabetes, BMI, dyslipidemia), ASCVD score, echocardiographic parameters (LVEF, DD, LVEDD, LVMI, E/A, Ea, E/Ea, and Ea/Aa), other laboratory parameters (Hb, UA, total and HDL cholesterol, TG), and other medical therapy (BB, CCB, statins).

**Table 1:** Baseline characteristics

Clinical data	Total IgAN group (n = 79)	Control group (n = 50)	P	IgAN CKD 1–2 (n = 62)	IgAN CKD 3–5 (n = 17)	P
Man/woman (n/%)	50/29 (63/37)	31/19 (62/38)	NS	37/25 (60/40)	13/4 (76/24)	NS
Age (year)	46.3 ± 11.2	45.8 ± 12.8	NS	43.8 ± 11.2	55.5 ± 7.7	< 0.001
Average systolic/diastolic RR (Hgmm)	122/73 ± 14/9	127/76 ± 13/9	0.04	120/72 ± 11/8	129/76 ± 16/9	0.014
24 h pulse pressure (Hgmm)	49.2 ± 10.7	51.2 ± 9.3	NS	48.3 ± 12.8	53.7 ± 7.7	0.048
Diurnal index systolic (%)	10.67 ± 5.6	9.1 ± 5.5	NS	11.7 ± 6.2	7.2 ± 5.2	0.004
**Metabolic parameters**						
Hypertension (n, %)	60 (76)	31 (62)	0.045	43 (69)	17 (100)	0.004
BMI (kg/m2)	28.1 ± 4.8	27.9 ± 5.1	NS	26 ± 4.7	28.4 ± 4.5	0.015
Dyslipidaemia (n, %)	36 (46)	26 (52)	NS	26 (42)	10 (59)	NS
Diabetes (n, %)	25 (32)	17 (34)	NS	17 (27)	8 (47)	NS
eGFR (ml/min)	87.2 ± 32.4	107.4 ± 18.3	< 0.001	98.2 ± 27.8	39.7 ± 29.6	< 0.001
Duration of kidney disease (year)	10.8 ± 9.4	-	-	10 ± 9	11.5 ± 10	NS
Smoking (n, %)	14 (18)	10 (20)	NS	10 (16)	4 (23)	NS
Metabolic syndrome (n, %)	22 (28)	15 (30)	NS	14 (22)	8 (47)	0.023
ASCVD risk score (%)	6.49	7.37	NS	4.63	7.10	NS
**Arterial stiffness parameters**						
Stiffness index (SI, m/s)	10.3 ± 2.59	8.88 ± 2.2	< 0.001	9.99 ± 2.13	10.97 ± 2.29	0.045
Pulse wave velocity (PWVao, m/s)	9.89 ± 1.65	8.6 ± 1.5	< 0.001	9.71 ± 1.61	10.72 ± 1.61	0.029
Aortic systolic blood pressure (SBPao, Hgmm)	121.1 ± 18.3	122.0 ± 19.3	NS	119.5 ± 16.4	127.9 ± 23.9	NS
Augmentation index (Aix)	-20.3 ± 29.0	-30.4 ± 28.2	0.049	-21.9 ± 29.9	-13.4 ± 23.6	NS
**Echocardiographic parameters**						
LVEF (%)	62.4 ± 6.5	63.4 ± 6.9	NS	62.2 ± 4.9	63.1 ± 7.7	NS
LVMI	106.6 ± 22.8	108.67 ± 21.1	NS	101.5 ± 16	125.2 ± 23	< 0.001
LVM (g)	204.4 ± 51.4	200.5 ± 49.1	NS	194.9 ± 44.0	239.0 ± 48.8	< 0.001
LVEDD (cm)	4.95 ± 0.4	4.83 ± 0.4	NS	4.93 ± 0.39	5.05 ± 0.41	NS
DD (n/%)	37 (47)	20 (40)	NS	24 (39)	13 (76)	0.003
E/A	1.05 ± 0.33	1.07 ± 0.38	NS	1.11 ± 0.32	0.85 ± 0.24	< 0.001
EDT (ms)	192 ± 40.6	186.7 ± 40.4	NS	185.8 ± 33.8	215.3 ± 43.5	0.003
Ea (cm/s)	13.2 ± 3.69	13.3 ± 5.0	NS	14.0 ± 2.13	10.5 ± 2.08	< 0.001
Aa (cm/s)	11.3 ± 3.0	11.1 ± 3.0	NS	11.0 ± 3.41	12.3 ± 2.63	NS
Ea/Aa	1.28 ± 0.57	1.32 ± 0.69	NS	1.38 ± 0.54	0.91 ± 0.32	< 0.001
E/Ea	4.88 ± 1.31	4.08 ± 1.54	NS	4.72 ± 0.95	5.43 ± 1.40	0.024
LVR	0.94 ± 0.25	1.04 ± 0.33	NS	0.94 ± 0.24	0.97 ± 0.26	0.035
**Laboratory results**						
Hb (g/dl)	13.6 ± 1.53	13.8 ± 1.42	NS	13.8 ± 1.56	13.1 ± 1.54	NS
MAU (mg/day)	484.6 ± 658.4	35.9 ± 30.9	< 0.001	420.8 ± 550.9	717.3 ± 721.8	0.016
HUS (umol/l)	320.5 ± 76.7	315.4 ± 65.8	NS	307.9.4 ± 76.7	366.8 ± 68.8	0.015
Total cholesterol (mmol/l)	5.03 ± 1.21	4.95 ± 1.20	NS	5.05 ± 0.95	5.16 ± 1.41	NS
HDL cholesterol (mmol/l)	1.28 ± 0.51	1.25 ± 0.45	NS	1.32 ± 0.64	1.15 ± 0.36	NS
TG (mmol/l)	1.69 ± 1.05	1.86 ± 1.10	NS	1.60 ± 0.90	2.00 ± 1.12	NS
**Therapy**						
ACEI/ARB (n, %)	65 (82)	27 (54)	0.003	45 (72)	14 (82)	NS
BB (n, %)	22 (28)	19 (38)	NS	10 (16)	3 (18)	NS
Statin (n, %)	26 (33)	21 (42)	NS	12 (19)	4 (23)	NS
CCB (n, %)	22 (28)	17 (34)	NS	12 (19)	4 (23)	NS

BMI: body mass index; eGFR: estimated glomerular filtration rate; ASCVD: atherosclerotic cardiovascular disease risk score; ACEI:angiotensin converting enzyme inhibitor; ARB: angiotensin receptor blocker; BB:beta-blocker; CCB:calcium channel blocker; CAD: coronary artery disease; LVEF: left ventricule ejection fraction; LVMI:left ventricular mass index; LVM: left ventricular mass; DD:diastolic dysfunction; E/A:mitral inflow; EDT: mitral inflow E wave deceleration time; Ea: early diastolic transmitral pulse-wave Doople flow; Aa: late (atrial) transmitral pulse-wave Doppler flow; Hb:hemoglibin; MAU:micro E/A: erly and late mitral inflow; EDT: E wave deceleration time; Ea: early diastolic velocity; Aa: late diastolic velocity; LVR: left venmtricular rigidity. albuminuria; HUS: uric acid; HDL: high-density lipoprotein; TG: triglyceride

Based on eGFR, IgAN patients were separated into two categories (CKD 1–2 vs. CKD 3–5). The baseline characteristics showed significant differences in age, blood pressure, metabolic parameters (hypertension, BMI), eGFR, and arterial stiffness parameters (SI, PWVao), as well as conventional echocardiography-measured parameters (E/A, EDT), tissue Doppler parameters (Ea, E/Ea, and Ea/Aa), and left ventricle rigidity (LVR). There was no significant difference in ASCVD score, LV ejection fraction, LV end-diastolic diameter, hemoglobin, lipid levels, RAAS blocker usage, or central aortic systolic blood pressure (SBPao) between the two groups. But there were significantly higher diastolic dysfunction, proteinuria, and uric acid levels in the CKD 3–5 group (Table 1).

**Fig. 1:**
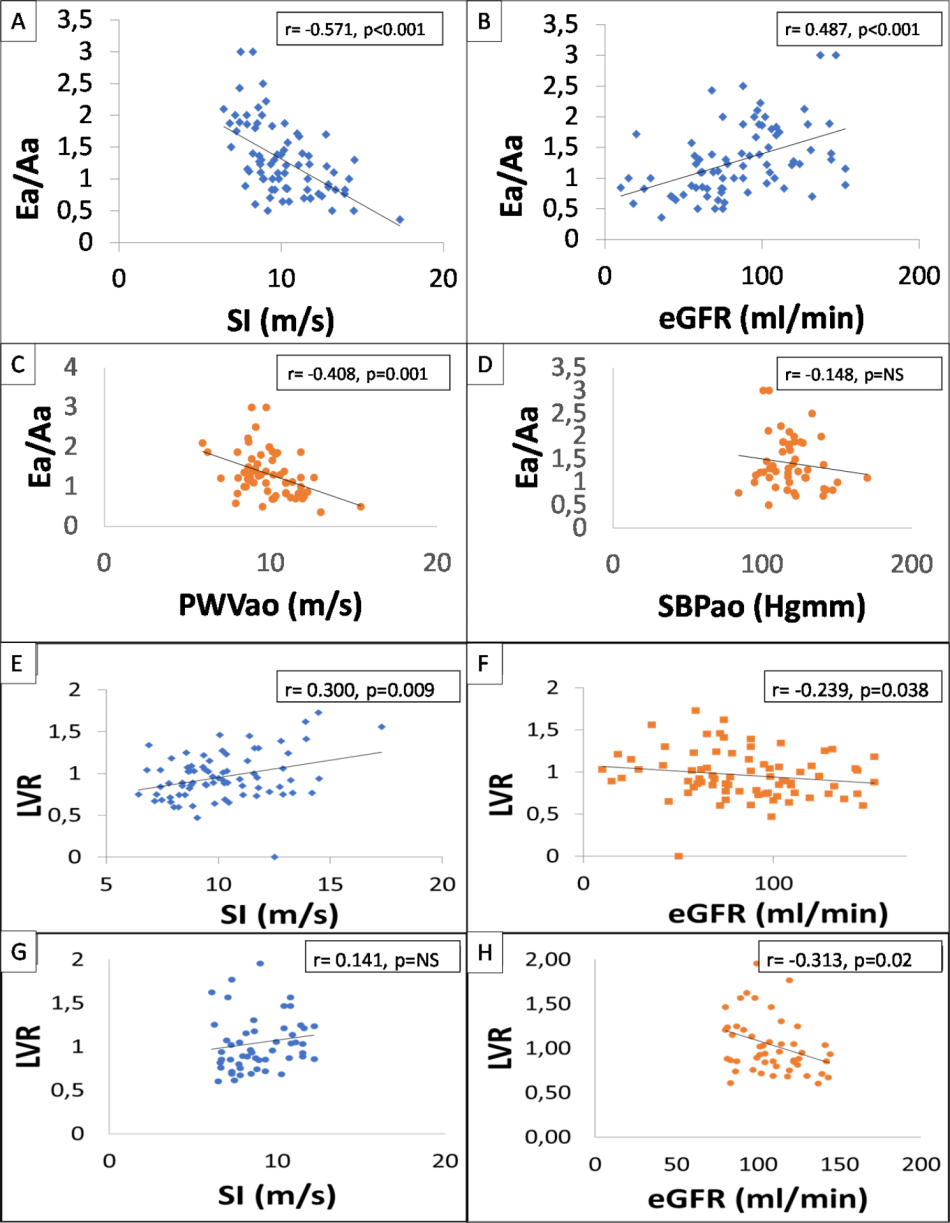
Correlation between Ea/Aa, and SI (**A**), eGFR (**B**), PWVao (**C**) and SBPao (**D**) in IgAN and correlation between LVR, SI (**E**) and eGFR (**F**) in IgAN and in the control group (**G**-**H**)

In the CKD 3–5 group, the incidence of diastolic dysfunction increased significantly: 39% vs. 76% (p = 0.003). There was a strong negative correlation between Ea/Aa and SI (r = -0.571; p < 0.001), also with PWVao (r = -0.408; p < 0.001), and a positive correlation between Ea/Aa and eGFR (r = 0.487; p < 0.001) in IgAN patients (Fig. 1A-D), with the same results in the control group for SI (r = -0.466; p = 0.005), eGFR (r = 0.373; p = 0.01), and PWVao (r = -0.492; p = 0.003). LVR was significantly correlated positively in IgAN patients with SI (r = 0.300; p = 0.009), but not in the control group (r = 0.141; p = NS), and significantly negatively with eGFR (r = -0.239; p = 0.038), similarly to the control group (r = -0.313; p = 0.02) (Fig. 1E-H).

There was a significant relationship between arterial stiffness parameters (SI, PWVao) and diastolic function parameters: mitral inflow E and A wave ratio (E/A) (SI vs. E/A: r = -0.441; p = 0.01; and PWVao vs. E/A: r = -0.410; p = 0.01), and E wave deceleration time (EDT) (r = 0.369). There was also a significant correlation between eGFR and diastolic function parameters: E/A (r = 0.466; p < 0.001), EDT (r = -0.363; p = 0.001), and tissue Doppler image parameters: Ea (r = 0.544; p = 0.001), E/Ea (r = -0.270; p = 0.017), and Ea/Aa (r = 0.455; p = 0.001) (Table 2).

**Table 2:** Correlation

	SI		PWVao		eGFR		SBPao	
	r	p	r	p	r	p	r	p
E/A	-0.405	< 0.001	-0.410	0.001	0.466	< 0.001	-0.347	0.006
EDT	0.223	NS	0.182	NS	-0.363	0.001	0.192	NS
Ea	-0.550	< 0.001	-0.287	0.024	0.544	< 0.001	-0.123	NS
E/Ea	0.355	0.002	0.385	NS	-0.270	0.017	-0.060	NS
Ea/Aa	-0.571	< 0.001	-0.419	0.001	0.455	< 0.001	-0.148	NS
LVR	0.300	0.009	0.048	NS	-0.310	0.013	-0.072	NS

E/A: erly and late mitral inflow; EDT: E wave deceleration time; Ea: early diastolic velocity; Aa: late diastolic velocity; LVR: left ventricular rigidity

**Fig. 2:**
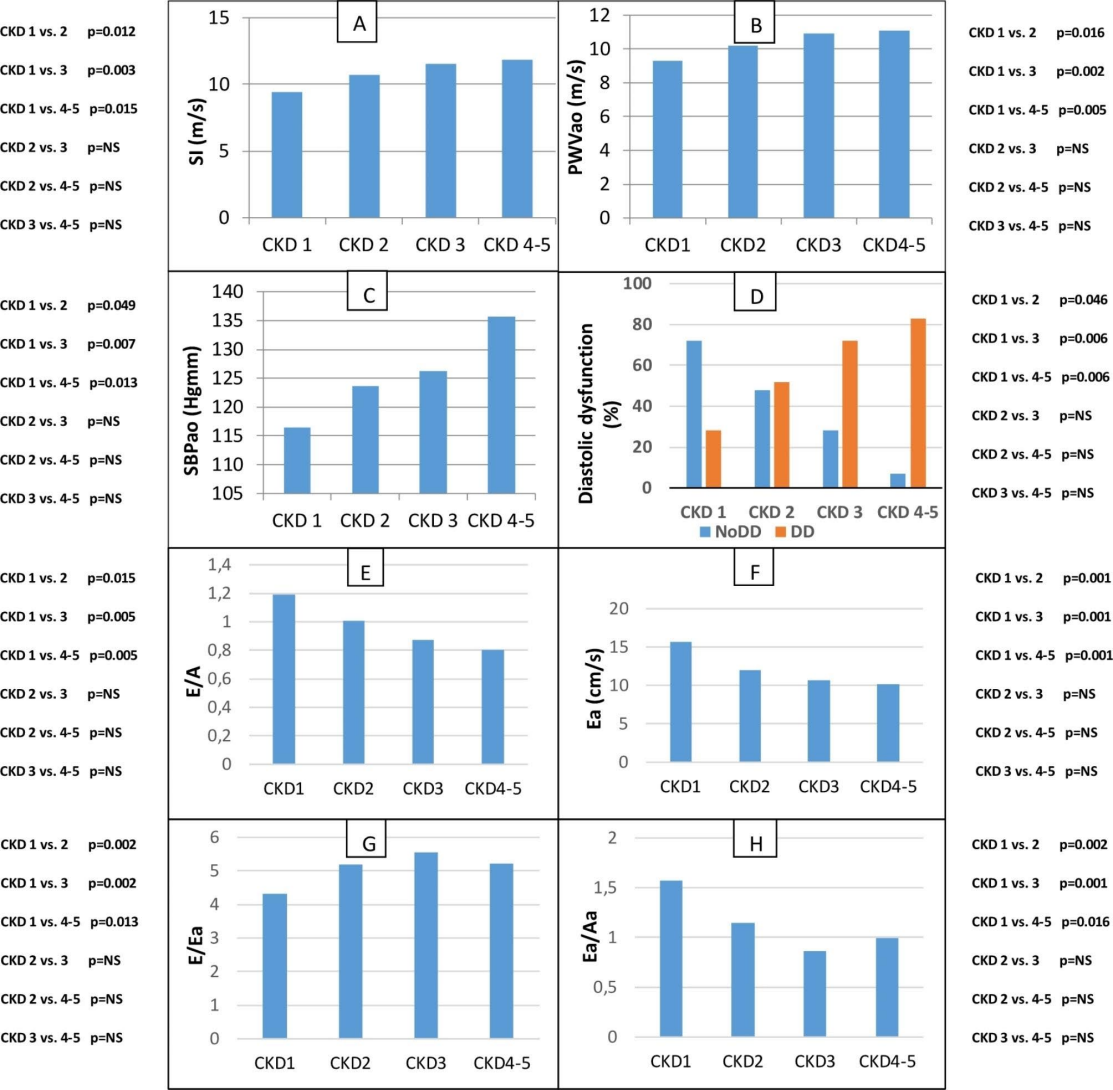
Differences in CKD stages (CKD 1 vs. CKD 2 vs. CKD 3 vs. CKD 4–5) in SI (**A**), PWVao (**B**), SBPao (**C**), diastolic dysfunction occurance (**D**), E/A (**E**), Ea (**F**), E/Ea (**G**) and Ea/Aa (**H**) in IgAN.

In different stages of CKD (CKD 1 vs. CKD 2 vs. CKD 3 vs. CKD 4–5), there were significantly higher SI, PWVao, SBPao, and diastolic dysfunctions associated with the deteriorating renal function, but there was no significant difference between the advanced stages. There was significantly higher E/Ea (CKD 1 vs. 2, p = 0.002, CKD 1 vs. 3, p = 0.002; CKD 1 vs. 4–5, p = 0.013; CKD 2 vs. 3, p = NS; CKD 2 vs. 4–5, p = NS; CKD 3 vs. 4–5, p = NS) and lower Ea/Aa (CKD 1 vs. 2, p = 0.002, CKD 1 vs. 3, p = 0.001; CKD 1 vs. 4–5, p = 0.016; CKD 2 vs. 3, p = NS; CKD 2 vs. 4–5, p = NS; CKD 3 vs. 4–5, p = NS) in the early stages of CKD (see Fig. 2).

**Fig. 3:**
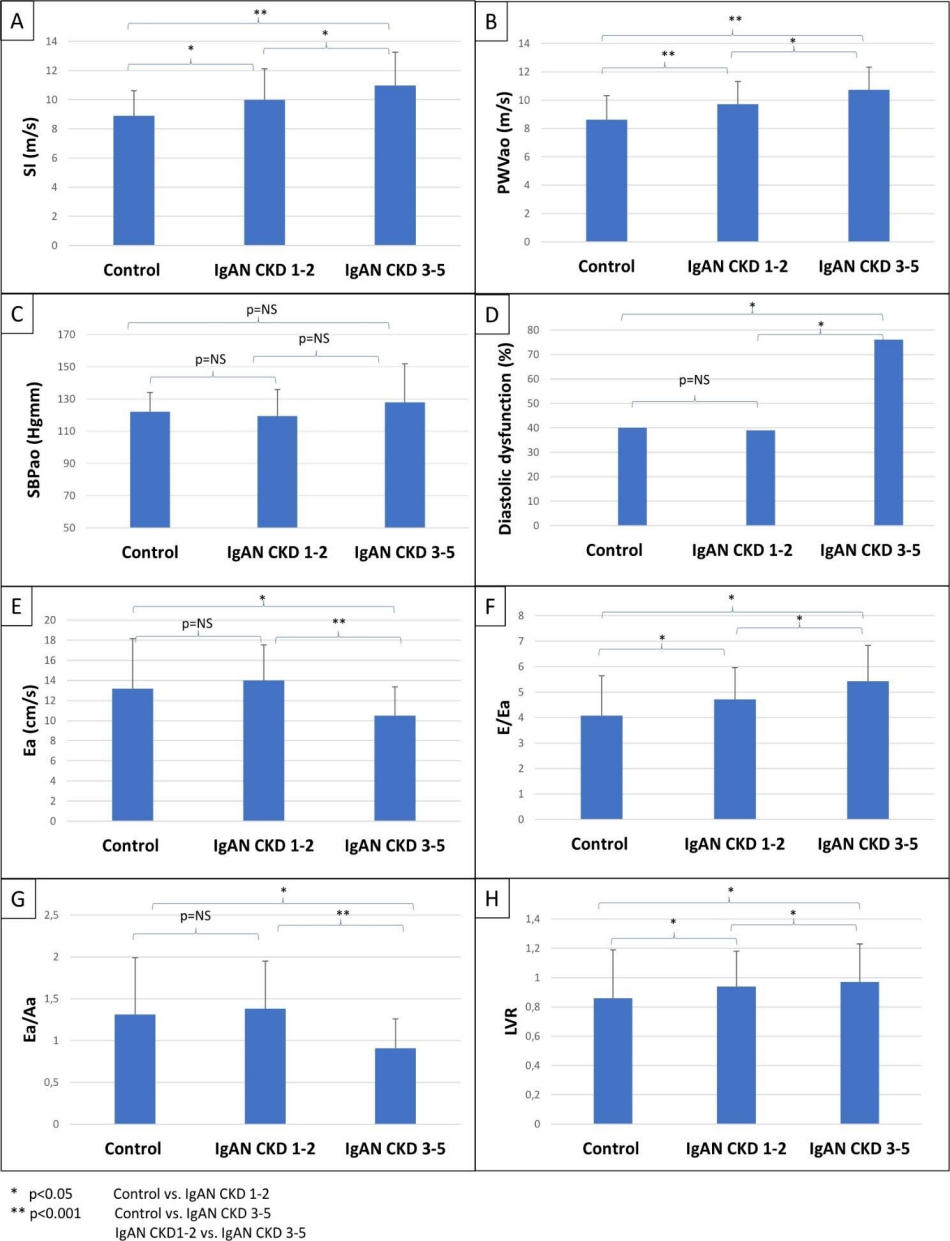
Differences in IgAN CKD 1–2 vs. CKD 3–5 and control group in SI (**A**), PWVao (**B**), SBPao (**C**), diastolic dysfunction occurrence (**D**), Ea (**E**), E/Ea (**F**), Ea/Aa (**G**) and LVR (**H**)

When we compared the IgAN groups (CKD 1–2 vs. CKD 3–5) to the control group, we found significantly higher SI (control vs. IgAN CKD 1–2, p = 0.001; control vs. IgAN CKD 3–5, p < 0.001; IgAN CKD 1–2 vs. IgAN CKD 3–5, p = 0.045) and PWVao (control vs. IgAN CKD 1–2, p < 0.001; control vs. IgAN CKD 3–5, p < 0.001; IgAN CKD 1–2 vs. IgAN CKD 3–5, p = 0.029) in the IgAN groups. There was no significant difference in SBPao between the groups. Diastolic dysfunction occurred significantly higher in the IgAN CKD 3–5 group compared to the control (p = 0.004) and the IgAN CKD 1–2 group (p = 0.003). The E/Ea ratio and the LVR increased significantly between the control vs. IgAN CKD 1–2 (p = 0.014, p = 0.047); control vs. IgAN CKD 3–5 (p = 0.025, p = 0.045); and between IgAN CKD 1–2 vs. IgAN CKD 3–5 (p = 0.024, p = 0.035) groups. The Ea and the Ea/Aa ratio decreased significantly in the IgAN CKD 3–5 group compared to the control (p = 0.018, p = 0.011) and IgAN CKD 1–2 groups (p < 0.001, p < 0.001) (Fig. 3).

**Fig. 4:**
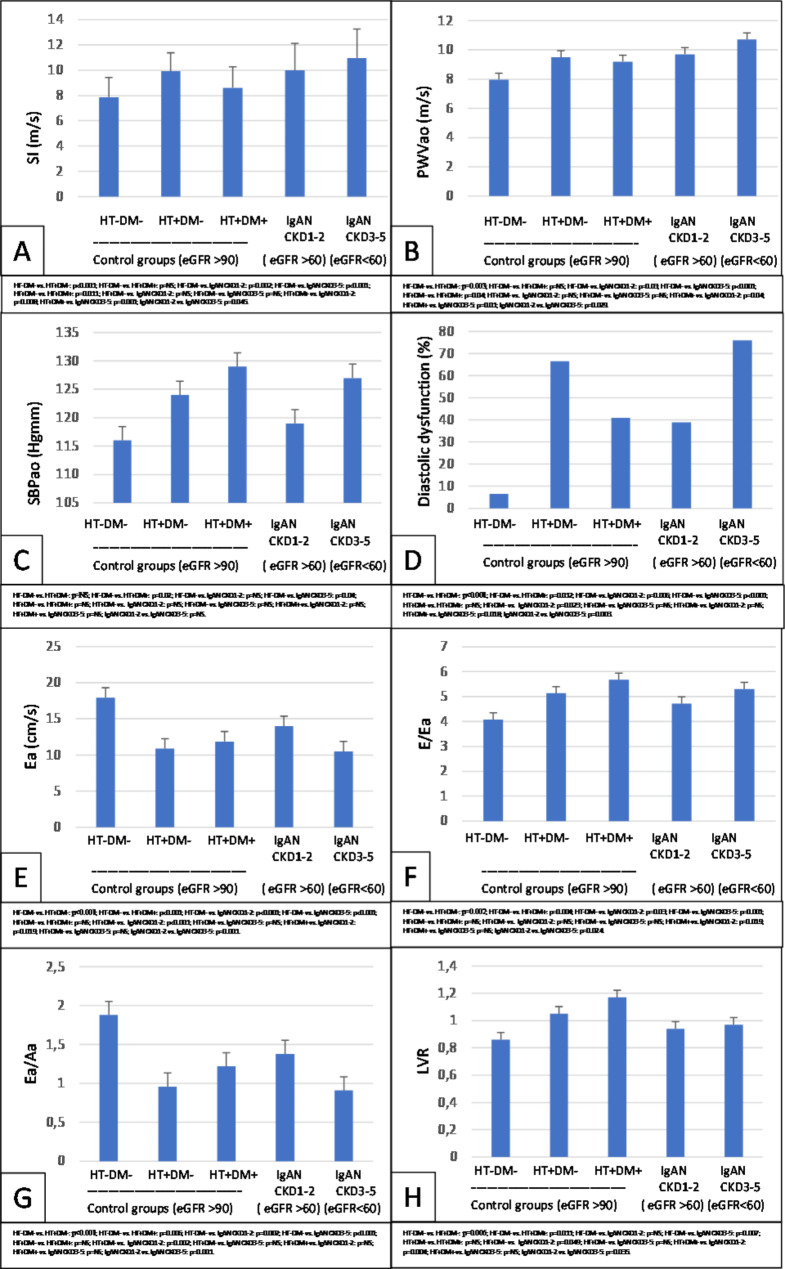
Subgroup analysis: Differences in IgAN CKD 1–2 vs. CKD 3–5 and control group (HT-DM- vs. HT + DM- vs. HT + DM+) in SI (**A**), PWVao (**B**), SBPao (**C**), diastolic dysfunction occurance (**D**), Ea (**E**), E/Ea (**F**), Ea/Aa (**G**) and LVR (**H**) HT: hypertension; DM: diabetes mellitus; IgAN: Immunglobulin A nephropathy; CKD: chronic kidney disease; SI: stiffness index; PWVao: aortic pulse wave velocity; DD: diastolic dysfunction; Ea: early diastolic velocity; Aa: late diastolic velocity; LVR: left ventricular rigidity

Subgroup analysis was performed to compare the CKD 1–2 and CKD 3–5 stages IgAN patients and the preserved renal function control group patients with and without hypertension and diabetes (HT-DM- (n = 15); HT + DM- (n = 18); HT + DM+ (n = 17)). We found a significant increase in SI (p = 0.002; p < 0.001), PWVao (p = 0.03; p < 0.001), E/Ea (p < 0.002; p < 0.001) and LVR (p = NS; p = 0.007), but a reduction in Ea (p < 0.001; p < 0.001) and Ea/Ea (p = 0.03; p = 0.001) compared both IgAN groups (CKD 1–2 and CKD 3–5) to a non-hypertensive, non-diabetic group with preserved renal function (Fig. 4).

By linear regression analysis, the independent predictors of SI were age, E/A, and E/Ea, and the independent predictors of PWVao were age and Aa. By linear regression analysis, the LVR independent predictor was only SI, and the eGFR predictors were E/A and hypertension. SBPao’s independent predictor was only E/A, but E/A independent predictors were age and eGFR; furthermore, Ea/Aa independent predictors were age and SI (Table 3).

**Table 3:** Linear regression analysis

Dependent variable	Independent predictor	B	95% Conf. Int. Lower	95% Conf. Int. Upper			p
SI	Age	0.065	0.021	0.108			0.004
	E/Ea	0.657	0.346	0.969			< 0.001
	E/A	-1.511	-2.918	-0.104			0.036
PWVao	Age	0.211	0.088	0.334			0.001
	Aa	0.047	0.012	0.082			0.009
SBPao	E/A	-19.630	-33.313	-5.947			0.006
LVR	SI	0.044	0.015	0.072			0.003
eGFR	HT	-21.110	-37.357	-4.863			0.012
	E/A	29.428	6.671	52.186			0.012
E/A	Age	-0.015	-0.022	-0.008			< 0.001
	eGFR	0.003	0.001	0.005			0.029
E/Ea	SI	-0.100	-0.161	-0.040			0.002
Ea/Aa	Age	-0.019	-0.032	-0.006			0.004
	SI	0.264	0.132	0.396			< 0.001

SI: stiffness index; PWVao: pulse wave velocity of the aorta; SBPao: systolic blood pressure of the aorta; LVR: left ventricle rigidity; eGFR: estimated glomerular filtration rate; E/A: early and late mitral velocity; Aa: late diastolic velocity; E/Ea: LV filling pressure; Ea/Aa: early/late diastolic velocity; HT: hypertension

## Discussion

Our study examined the relationship of conventional Doppler and tissue Doppler echocardiography parameters with renal function and arterial stiffness parameters in a homogenous, immunocomplex-mediated CKD population of IgAN patients. We found a correlation between Ea/Aa, and eGFR, SI, and PWVao in the IgAN group and the same in the control group in the cases of SI, PWVao, and eGFR, but not with SBPao.

Based on our results in IgAN patients, the increase in diastolic dysfunction is observed by decreasing renal function, which is best described by E/A, EDT, and Ea as defined by TDI, similarly in CKD, which could have a close relation to arterial stiffness.

Cardiovascular alterations (based on echocardiographic parameters) develop in the early stages of CKD (which was most pronounced in the IgAN CKD 3–5 compared to non-CKD controls). There may be a difference in the dynamics of these, which may stem from the etiology of the disease [21], but there is a small amount of data in the literature on this. Patients at high CV risk should be screened in the early stages of CKD. Based on our results, the TDI parameters are sensitive early markers, supported by a close correlation with eGFR, even in a relatively low number of cases. According to our data, early in IGAN, with still preserved kidney function, changes in the heart and blood vessels begin in some patients.

Several previous studies have found an association between LV diastolic function and arterial stiffness in normal subjects. Cauwenberghs et al. studied 1233 subjects from the general population and showed that diastolic parameters are significantly correlated with cfPWV and central pulse pressure as measured by arterial tonometry [9]. We found similar results using a photoplethysmographic and oscillometric method in IgAN and our control group as well. The Framingham Heart Study, a recent large study with 5799 participants, likewise found strong relationships between cfPWV and central pulse pressure, lateral e’ velocity, and E/e’ [30]. In a small study with 58 subjects utilizing applanation tonometry, Borlaug et al. demonstrated that the carotid augmentation index and carotid characteristic impedance have an independent relationship with LV septal e’ velocity [31]. We found similar correlations in IgAN and control patients between SI, PWVao, and Ea.

Previous studies have shown that E/Ea, an estimate of LV filling pressure by Doppler echocardiography, is a predictor of all-cause mortality in patients with LV systolic dysfunction and after acute myocardial infarction [32, 33]. Another study in patients with ESRD also reported that an E/Ea ≥ 15 could predict an increase in LV filling pressure with a sensitivity of 82% and specificity of 88% and was associated with an increased risk of mortality [34]. In addition to predicting all-cause mortality, a high E/Ea has been reported to provide additional prognostic value in patients with ESRD beyond traditional echocardiographic parameters [34, 35]. A high E/Ea ratio may lead to high volume status, increase renal efferent pressure, and decrease renal blood flow, subsequently leading to a progressive decline in renal function [36, 37]. A higher preload status may also contribute to a more rapid progression to dialysis. In this cohort in the early stages of CKD, there was a significant increase in E/Ea but not in the CKD 4–5 stage group, which may indicate that compensatory mechanisms are beginning to be exhausted, but the central aortic blood pressure, arterial stiffness, and left ventricle rigidity were higher in the CKD 4–5 group. Similar alterations were observed in our IgAN patients compared to the controls but can be observed in earlier stages of CKD.

In CKD, several conditions contribute to the pathogenesis of HFpEF, of which the central aortic blood pressure may be the most important such as peripheral arterial hypertension [38]. Left ventricular hypertrophy (LVH) is likewise one of the foremost myocardial modifications in CKD, and CKD itself performs a crucial position in its development. It develops early in the progression of kidney dysfunction and is frequently accompanied by myocardial fibrosis and LVDD. LVH is an independent risk factor for mortality in this population as well. The role of CKD is well documented but not fully elucidated in terms of its basis. The effect of uremia on the myocardium includes structural changes such as cardiomyocyte hypertrophy, myocardial fibrosis, and thickening of the intramural arteries. Together, these structural changes predispose to LVDD in response to the cumulative action of traditional and CKD-related risk factors [39–41]. There is good evidence that interstitial fibrosis is related to changes in collagen myocardial metabolism. On the other hand, cardiomyocyte hypertrophy and vascular remodeling may be adaptive responses to pressure and volume overload [42]. But other factors, such as hyperphosphatemia, hyperparathyroidism, and hypovitaminosis D, are more common in advanced CKD and dialysis patients [43, 44]. Another important factor is the renin-angiotensin-aldosterone system (RAAS) activation, which potentially induces myocardial fibrosis and hypertrophy. Activation of the intracardiac RAAS seems to be critically involved in the overload status observed in dialysis, but angiotensin II and aldosterone can also be involved in myocardial cell hypertrophy and fibrosis independent of afterload [45].

In Escoli et al.’s review, they suggest patients with CKD and ESRD should be monitored regularly (perhaps every 1–2 years) for the development and assessment of the severity of LVH and cardiac fibrosis, most likely with serial echocardiography [46]. Nonetheless, our findings suggest that it may also be beneficial for CKD stages 1–3 in high-risk patients. The intensity of renal function deterioration may be a more important factor than the existence of CKD. In our patients, the high rate of RAAS inhibition was carried out in accordance with the guidelines. Finerenone appears to be a promising molecule for inhibiting the progression of renal function and reducing blood pressure [47], proteinuria [48, 49], and mortality [50], especially in diabetic patients with CKD.

Hypertension is well known to be a major risk factor for the development of LVDD in patients on chronic hemodialysis [51]. However, in the early stages of CKD, when the blood pressure elevation is not so significant, the mechanism of the relationship between CKD and LVDD is not fully characterized. Therefore, the value of the echocardiography examination should be important in CKD. The factors of progression in IgAN are not specific; they are similar to those in CKD. CKD risk factors overlap to a large extent with CV risk factors but may add an extra CV risk as well.

In Shu’s KNOW-CKD study, LVDD was independently associated with adverse CV outcomes and all-cause mortality in patients with predialysis CKD [52]. Liang et al. proved that systolic dysfunction and LVDD demonstrated mutually augmentative effects on CV mortality and suggested that, along with traditional nephroprotection, early cardioprotection should be given priority in CKD patients. As a result, after CKD diagnosis, cardioprotective intervention should be started as soon as possible [53].

The acceleration of renal function loss and progression to ESRD are both worsened by known CV risk factors, such as baseline eGFR, proteinuria, and hypertension, which are also risk factors for CKD progression. However, the progression of CKD, a complex process, cannot be explained in all cases by these traditional risk factors. This may also underline the importance of arterial stiffness measurement.

Similar findings were made by Redfield et al. [39], who examined 2042 members of the community. They found that female gender and advanced age are associated with elevated arterial and LV diastolic stiffness. Our results support their studies, demonstrating a pronounced association between SI, PWVao, and diastolic indices. Widely different study populations and measurements with various vascular stiffening sensitivities may contribute to the divergent results between investigations.

LVDD is closely linked to arterial stiffness because the uremic milieu predisposes patients with CKD to systemic arterial stiffness and myocardial interstitial fibrosis, ultimately leading to LVH and impaired LV relaxation and compliance [54, 55]. Zanoli et al. demonstrated that the large arterial stiffening starts early during CKD, even in patients with a very mild reduction of renal function (GFR 60–89 ml/min per 1.73 m2 without proteinuria) and that this alteration precedes the arterial wall remodeling [56]. We can also confirm that in the early stage of IgAN (CKD 1–2) where the stiffening (PWV) was significantly higher than in the controls.

Our data revealed that hypertension is a highly common consequence in IgAN [57, 58], affecting 50–70% of patients, it was even slightly higher among our IgAN pts (76%). Vascular events are more common in CKD due to elevated RAAS activity and hypertension. Thus, RAAS blockade is the standard treatment (recommended in all guidelines) for these patients in general and also for those who have IgAN [59, 60]. Based on our previous results and those of others, we thought that RAAS also plays a key role in the development of arterial stiffness and LVDD in renal disease, as in IgAN [61]. However, there is no data on whether ACEI and/or ARB treatment could afford an LVDD-lowering effect in patients with IgAN. In our study, more patients received ACEI and/or ARB therapy in the lower eGFR group than in the higher group. But we were not able to analyze users and non-users of RAAS inhibitor treatment. In our study, there was no significant difference in the use of a RAAS inhibitor between two different renal function groups (CKD 1–2 vs. CKD 3–5). Based on this observation, RAAS may be important in evaluating LVDD. However, it should be noted that the blood pressure of the study population was well controlled, but there were significantly higher incidences of hypertension and a higher BMI in the CKD 3–5 group than in CKD 1–2 in IgAN, which could call attention to these CKD patients’ optimal blood pressure and reduce their body weight to the optimal range to reduce the CV risk. But despite the RAAS blockade arterial stiffness showing an increase already in CKD 1–2, RAAS blockade could not prevent its increase.

Similar results were also found in a population of rheumatoid arthritis patients in which inflammation is the main component [62]. In the case of IgA nephropathy, the renal disease itself could cause subclinical inflammation (as we can see in the renal biopsy specimens), which the decreasing GFR could further aggravate.

In the case of subgroup analysis, these accompanying factors such as hypertension and diabetes further worsen the patients’ CV risk, so special attention should be paid to them.

Our results may highlight the potential contribution of increased pulsatile load and LV-arterial coupling to LV diastolic dysfunction in CKD. Given that LVDD is a main determinant of HFpEF, it can also be postulated that increased arterial stiffness is an important risk factor for developing HFpEF. In addition, arterial stiffness could be a good monitoring tool to assess LV diastolic dysfunction. Measuring arterial stiffness in routine clinical practice is a cheaper and simpler test than echocardiography, but should be used with due caution. The prevention of heart failure or, more importantly, a delay in the evolution of LV diastolic dysfunction may be good therapeutic goals for decreasing arterial stiffness.

### Limitations of the study

Our results showed that the values of tissue Doppler parameters and arterial stiffness can be useful for clinical evaluation. However, difficulties may arise during echocardiographic and arterial stiffness measurements. In some cases, specifically with the elderly, a lack of cooperation can be a problem.

The arterial stiffness measurement is determined by two non-gold standard methods first is finger photoplethysmography, second an oscillometric method. Occasionally, there are difficulties in registering the digital pulse volume and pulse wave velocity. It may be problematic in some cases, mostly in elderly patients, to confidently separate systolic and reflective waves and thus assess the stiffness index. Atrial fibrillation and frequent atrial and ventricular ectopic activity could also limit correct pulse curve detection. We estimated and did not measure renal function. However, the use of eGFR is widely accepted throughout the literature. Data analyses may be weakened by the low number of our cases and the relatively low number of patients with CKD 4–5. The current study failed to look at the degree or progression of proteinuria. The evaluation of the results may also be weakened by the low number of female patients. We did not examine left atrial volume, myocardial strain, or strain rate. In our study, we examined only one etiology of CKD, IgAN.

Despite these limitations, the results of this study highlight that the onset of target organ damage in CKD is predicted by TDI and arterial stiffness.

## Conclusion

Our results suggest that the simultaneous use of arterial stiffness and tissue Doppler echocardiography parameters (Ea and E/Ea) appears to be a suitable process to identify high-risk, asymptomatic CKD patients. Arterial stiffness elevation compared with a control group can indicate an increase in CV risk at an earlier stage of CKD.

Lower Ea and higher E/Ea, PWV, or SI should call attention to those CKD patients who have higher renal and CV risk and need to be monitored more closely, referred for further CV tests, and given maximal nephroprotection. It is not the duration of the kidney disease but its progression and the loss of kidney function that is dangerous.

Our findings support the role of tissue Doppler echocardiography and arterial stiffness measurement in the high-CV-risk population of CKD patients, which also helps to understand the relationship between heart abnormalities and renal impairment.

In conclusion, impaired renal function gradually correlates with Ea, E/Ea, SI, and PWV parameters in patients with CKD. Decreased renal function is associated with decreased Ea and increased E/Ea, which could be responsible for a later poorer prognosis due to worse CV and renal outcomes. In the background, the role of common vascular and myocardial pathological remodeling could be hypothesized, which is worsened by metabolic changes and immunological alterations in this group of patients.

Further large-scale, multicenter prospective studies are warranted to evaluate the role of CV risk factors in mediating the changes in the TDI and vascular stiffness parameters, as well as the complex relationships between CV disease and CKD.

## Data Availability

The datasets used and analyzed during the current study are available from the corresponding author on reasonable request. Permission to reproduce from other resources: N/A.
